# Parental supply of alcohol to Australian minors: an analysis of six nationally representative surveys spanning 15 years

**DOI:** 10.1186/s12889-016-3004-2

**Published:** 2016-04-14

**Authors:** Adrian B. Kelly, Gary C. K. Chan, Megan Weier, Catherine Quinn, Matthew J. Gullo, Jason P. Connor, Wayne D. Hall

**Affiliations:** Centre for Youth Substance Abuse Research, The University of Queensland, Brisbane, QLD 4072 Australia; Centre for Youth Substance Abuse Research, Queensland University of Technology, Brisbane, Australia; School of Medicine, The University of Queensland, Brisbane, Australia

**Keywords:** Adolescent, Alcohol, Parent, Parental supply, Nationally representative

## Abstract

**Background:**

Most adolescents begin alcohol consumption during adolescence, heavy alcohol use by adolescents is common, and alcohol-related harm amongst adolescents is a major public health burden. Parents are a common source of alcohol amongst adolescents, but little is known about how parental supply of alcohol has changed over recent years. This study examines national trends in parental supply of alcohol to adolescent children in Australia since 1998.

**Methods:**

Six Australian National Drug Strategy Household Surveys (1998–2013) yielded rates of parental supply of current and first ever alcohol consumed. Lifetime and current alcohol use were also estimated. The surveys were conducted for households across all Australian states and territories. Surveyed adolescents were aged 14–17 years (*N* = 7357, 47.6 % male). Measures included the reported source of currently consumed alcohol and first ever alcoholic beverage (parents/friends/others), lifetime alcohol use, number of standard alcohol units consumed on drinking days, and frequency of alcohol use. Corrected Pearson chi-squared tests were used to compare survey years.

**Results:**

There was a significant drop in parental supply of current alcohol use from 21.3 % in 2004 to 11.79 % in 2013 (*p* < .001). The lower prevalence of parental supply coincided with legislative changes on parental supply of alcohol to adolescents, but causality cannot be established because of the variation in the timing and reach of parental supply legislation, and small samples in some states. There were downward trends in adolescent experimentation, quantity and frequency of alcohol use across years, with the largest drop in alcohol use in 2010 and 2013.

**Conclusions:**

In Australia, there has been a substantial reduction in parental supply of alcohol to adolescents from 2010, and this factor may partially account for reductions in adolescent alcohol use.

## Background

In Western countries, alcohol use and misuse amongst adolescents is very common. For example, in England, 74 % of 15 year olds had consumed alcohol and 27 % had been intoxicated in the past four weeks [11]. In the United States, 68.2 % of 12th Graders (18–19 years of age) have consumed alcohol and 50 % report that they have been intoxicated [13]. In Australia, 74 % of 14 year olds and 90.9 % of 17 year olds have ever consumed alcohol. Consumption of more than 4 drinks on one day in the past 7 days occurs for 2.6 % and 18.5 % of 14 and 17 year old Australians respectively [29]. Adolescent alcohol use involves substantial risks, including alcohol-related injury and assault [17], early sexual debut [22], depression [6], adult alcohol abuse/dependence [20], and premature death [27]. Alcohol may also more adversely affect brain function in adolescents than adults [12].

In most Western industrialised countries there is no legislated minimum age for alcohol use. Age-related restrictions commonly relate to the purchase of alcohol (e.g., under 21 years of age in the United States, under 18 years of age in Australia and the UK), entry to licensed clubs and other venues, and consumption of alcohol in unlicensed venues or spaces. The high prevalence of alcohol use amongst minors indicates that adolescents frequently obtain alcohol from other sources, most often parents and/or peers [10]. Around 35-38 % of adolescents in Australia and England report that a parent supplied them with alcohol [7, 28, 30]. While parents frequently cite harm minimization as the rationale for supplying alcohol to their children [8, 15], there is little evidence this practice is protective. A review of 22 cross-sectional and longitudinal studies found that parental supply increased alcohol-related risks and had few, if any, protective effects [14]. Some Australian research suggests that effects of parental supply on adolescent alcohol use may be significant when alcohol use is unsupervised rather than supervised, but there is insufficient research on the moderating effects of supervision to draw firm conclusions [14]. In response to the high prevalence of adolescent alcohol use/misuse an parental supply of alcohol, national guidelines in Australia and the United Kingdom on alcohol use recommend that people under 18 years of age delay the initiation of alcohol use for as long as possible, and that people under 15 years of age should abstain from any alcohol use [5, 19]. Over the period 2010–2014, a number of Australian states legislated to make it a criminal offence for adults other than parents/guardians or those acting with the permission of parents to provide alcohol to persons under the legal age for alcohol purchase.

The aim of this study was to examine the prevalence of adolescent alcohol use and parental supply of alcohol to adolescents using nationally representative Australian survey data from 1998 to 2013. We focused on adolescents aged 14–17 years of age because these cover the years immediately prior to when alcohol purchase and licensed venue entry is legal in Australia. It is also the period when alcohol use and misuse often escalates [16]. We also explored the adolescents’ reports of who supplied their first ever alcoholic beverage. Parental influences on alcohol use tend to be stronger during early adolescence than for later periods [3], so parental provision of alcohol at this age may have longer-term significance.

## Methods

### Sample

The sample was drawn from tri-annual consecutive National Drug Strategy Household Surveys (NDSHS) conducted in 1998, 2001, 2004, 20010 and 2013. The NDSHS is conducted in all Australian States and territories, with an overall sample size of over 20,000 at each survey (except for 1998 where *n* = 10,340). For the present study, only data for participants aged 14–17 years of age were analyzed. The sample size for this age group, and demographic statistics, including age, gender, and socioeconomic advantage/disadvantage are presented in Table 1. There were no statistically significant differences in age and regional composition across the surveys. There were significant differences across the surveys in gender, socio-economic advantage/disadvantage, and country of birth (*ps* < .05). However, the differences were very small (Cramer’s *V* = 0.05 for socioeconomic advantage/disadvantage, 0.06 for country of birth, and 0.04 for gender) and the data were weighted to adjust for these imbalances during survey execution. The response rates for 14–17 year old participants ranged from 46 % to 56 % across surveys (Table 1) and these rates were comparable to other Australian surveys [29].

**Table 1:** Sample characteristics of adolescent participants from 1998 to 2013

	1998		2001		2004		2007		2010		2013	
	*N*	%	N	%	N	%	N	%	N	%	N	%
Total sample size	10340		26744		29445		23356		26648		23855	
Sample size of the age group 14-17	1062	10.27	1477	5.52	1852	6.29	1066	4.56	1075	4.03	825	3.46
Male	517	48.68	718	48.61	838	45.25	503	47.19	501	46.6	404	48.97
Age												
14	269	24.48	311	21.06	453	24.46	247	23.17	242	22.51	188	22.79
15	266	25.05	366	24.78	463	25.00	256	24.02	275	25.58	197	23.88
16	263	24.76	404	27.35	469	25.32	289	27.11	264	24.56	224	27.15
17	273	25.72	396	26.81	467	25.22	274	25.7	294	27.35	216	26.18
Regionality–living in major cities^a^	-		-		1157	62.47	694	65.1	676	62.88	542	65.70
Socioeconomic advantage^b^												
Least advantaged	-		246	16.66	296	15.98	171	16.09	171	15.91	143	17.33
2nd quintile	-		345	23.36	357	19.28	193	18.16	236	21.95	158	19.15
3rd quintile	-		234	15.84	348	18.79	188	17.69	185	17.21	141	17.09
4th quintile	-		228	15.44	433	23.38	250	23.52	226	21.02	185	22.42
Most advantaged	-		424	28.71	418	22.57	261	24.55	257	23.91	198	24.00
Australian born	933	87.85	1295	87.68	1657	90.99	911	89.05	936	90.00	702	85.30
Response Rate^c^		56		50		46		49		51		49

^*a*^The coding for area of residence in Australia was different in 1998 and 2001, and was therefore not comparable. ^b^This information was not available in the 1998 survey. ^c^This is the overall response rate of the survey

### Measures

#### Current source of alcohol

This was assessed by ‘Where do you usually obtain your alcohol now?’ The response options were *friend or acquaintance brother or sister*, *parent*, *spouse or partner*, *other relative*, *stole it*, *purchased it myself from retailer*, *other* and *can*’*t recall*. Participants were required to mark only one response. Data on the following endorsements were included in this analysis: *Friend or acquaintance*, *Parent*, *Brother or sister*, *Other relative* and *Purchased it myself from retailer*.

#### Source of first alcoholic beverage

Assessed by ‘Who supplied you with the first glass of alcohol you consumed?’ The response categories and format were the same as for *Current source of alcohol*.

#### Alcohol use

Four dimensions of alcohol use were assessed–lifetime prevalence of trying alcohol, lifetime prevalence of ever consuming a full serve of alcohol, frequency of alcohol use in last 12 months (Weekly/Less than weekly/Ex-drinker/Non-drinker), and number of standard drinks on a drinking day (‘On a day that you have an alcoholic drink, how many standard drinks do you usually have?’, Non-current drinker/2 or less/3-4/5 or more). For this age group, the measure of having 5 or more drinks in a drinking day was used as an approximate measure for drinking to intoxication [4].

#### Demographic variables

Socioeconomic advantage/disadvantage was based on Socioeconomic Indexes for Areas (SEIFA) scores [1] SEIFA scores are based on population census variables related to disadvantage, such as low income, low educational attainment, unemployment, and dwellings without motor vehicles. Regionality was derived from postcode and was coded as “major cities”, “inner regional”, “outer regional” and “remote and very remote”.

### Procedure

Each NDSHS was approved by the Australian Institute of Health and Welfare Ethics Committee. Access to these survey data by the Centre for Youth Substance Abuse Research was approved by the Australian Social Science Data Archive and by The University of Queensland Human Research Ethics Committee. For each NDSHS, households were randomly selected using a multi-stage stratified design based on statistical local areas [1], with oversampling for small geographical locations.

National surveys were conducted by an independent research company under the direction of the Australian Institute of Health and Welfare (AIHW). Interviewers were located across Australian States and Territories, and underwent training sessions prior to data collection. Data were predominately obtained through a ‘drop and collect’ method across the six surveys (60-100 %). Self-completion questionnaires were delivered and collected to/from households. Householders were provided with a letter from the Director of the AIHW and brochure describing the study, and the confidentiality and anonymity of participation. Participants were provided with a dedicated free call number that was set up and managed by the Australian Institute of Health and Welfare to deal with respondent concerns and queries, as well as another dedicated free call number managed by the government-appointed survey contractors. Signed consent (from parents/guardians) was only obtained for those under 16 years of age. If participants were 16 years old or older, signed consent was not required, and participants were taken to have given informed consent via letter/brochure scaffolding and completion of the questionnaire. If collection was not possible a pre-paid, pre-addressed envelope was provided and a follow-up reminder telephone call was made.

For the 1998, 2001, 2004 and 2007 surveys, data collection was augmented by face-to-face interviews and/or Computer-Assisted Telephone Interviews. For all methods the respondent was the household member who was aged at least 14 or older (1998–2001) or 12 years or older (2004–2013) next to have a birthday. Signed parent/guardian consent was obtained for persons under 16 years of age. Individual consent was required from respondents over the age of 16 years of age. In cases where questionnaires were not returned or could not be collected, a non-response was recorded. Across all survey years, response rates ranged from 46-56 % (see Table 1). Further information on design and methods of the NDSHS can be found elsewhere [2]. To address any potential disparity arising from the survey design or its implementation, and to align the samples with the Australian population, weights were applied to the data based on geographic stratum. All analyses were performed in Stata 13 using the *svy* command to account for the complex survey design [26]. Corrected Pearson chi-squared tests were performed to compare the prevalence statistics across different year of the survey.

## Results

**Fig. 1:**
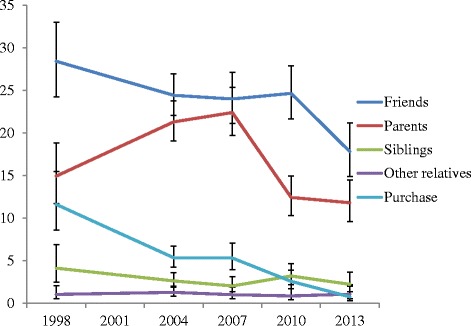
Top five sources of current alcohol consumed

Over most survey years, friends were the dominant current sources of alcohol (varying from 28.4 % in 1998 to 17.8 % in 2013) and parents the second most prevalent current source of alcohol (14.9 % in 1998 to 22.4 % in 2007). Compared to 1998, parental supply of alcohol increased in a statistically significant fashion from 1998 (14.9 % of the total sample) to 2004 and 2007 (21.3 % and 22.4 % respectively for the total sample) before decreasing to 11.8 % of the total sample in the 2013 survey (see Fig. 1). The overlap of confidence intervals for parental supply in 1998 and 2013 suggested a return to 1998 levels. Friends’ supply of alcohol showed a downward trend from 27.9 % in 1998 to 20.2 % in 2013. There was no significant change in the rate of friend’s supply of alcohol from 1998 to 2010 but this rate dropped significantly in 2013 (from 24.7 % in 2010 to 17.8 %). There was a large decrease in the rate of (illegal) purchase of alcohol over the six surveys from 11.6 % in 1998 to 0.7 % in 2013 (*p* < .05) (see Table 2). Overall, there was a reduction in the supply of alcohol from friends and parents from 2007, and reductions in parental supply appeared more substantial than reductions in supply by friends over this period.

**Fig. 2:**
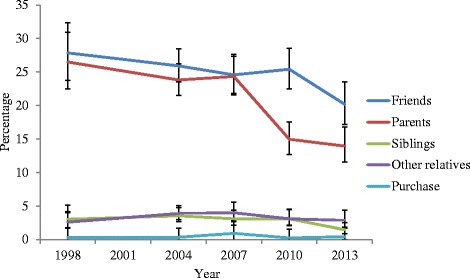
Top five sources of first alcohol beverage consumed

Adolescents reported that friends and parents were the sources of their first alcohol beverage, with very similar prevalence rates from 1998 to 2007 (range 24.3 % to 27.9 %). Siblings and other sources were comparatively rare as a source of first alcoholic beverage. In 2010 and 2013 there was a substantial and significant drop (*p* < .05) in the proportion of adolescents reporting that parents supplied their first alcoholic beverage–from 24.3 % in 2007 to 15.0 % and 13.9 % in 2010 and 2013 respectively (see Fig. 2). There was no evidence that the decrease in parental supply was compensated for by another source of first alcoholic beverage. Friends as the source of first alcoholic beverage did not differ between 2010 and previous surveys (around a quarter of the sample across years), and there was a significant drop in this rate in 2013 (from 25.4 % in 2010 to 20.2 %, *p* = .020). The rate of obtaining the first alcoholic beverage from siblings did not differ in 2010 compared to previous surveys but there was a significant drop in this rate in 2013 (from 3.09 % to 1.45 %, *p* = .024). There was no meaningful change in the prevalence of obtaining alcohol from other relatives and retail outlets.

**Fig. 3:**
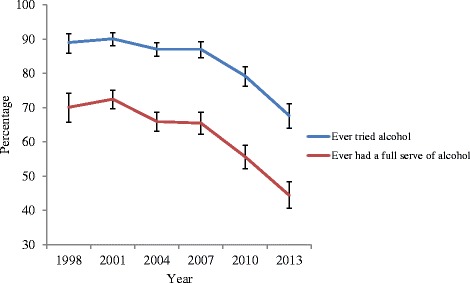
Trends in life time abstinence

**Fig. 4:**
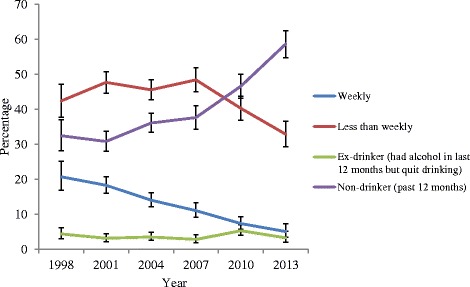
Trends in alcohol use in past 12 months

**Fig. 5:**
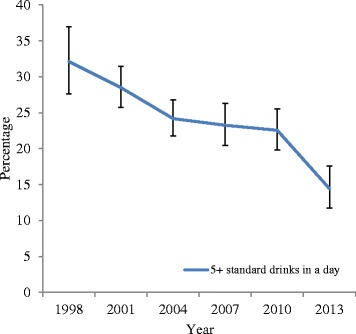
Trends for 5+ standard drinks a day

Corresponding trends in self-reported alcohol use are presented in Fig. 3. There were substantial decreases in lifetime alcohol use (ever tried any, ever had a full serve, see Fig. 3). From 1998 to 2007, the prevalence of ever tried alcohol was 87-90 %. This dropped statistically significantly in 2010 (79.3 %) and 2013 (67.8 %). There were significant and substantial drops in lifetime prevalence rates for having had a full serve of alcohol from 70.1 % in 1998 to 44.4 % in 2013 (*p* < .05). The frequency of alcohol use also dropped significantly and substantially between 1998 and 2013 (Fig. 4). In particular, the prevalence of weekly drinking decreased from 20.7 % to 5.1 % in this period. This decrease was accompanied by a significant drop in the rate of heavy drinking, defined as having 5 or more drinks in a day, which dropped from 32.1 % in 1998 to 14.4 % in 2013 (Fig. 5).

A supplementary analysis was performed to examine changes in parental supply of alcohol through logistic regression. Whether the adolescent obtained alcohol from the parent was regressed on survey year, controlling for age, gender and birthplace. Results from this analysis indicated that compared to 2004, adolescents in 2010 and 2013 were significantly less likely to obtain alcohol from parents (OR = 0.50, *p* < .001 for 2010 and OR = 0.49, *p* < .001 for 2013).

## Discussion

The present study used data from nationally representative surveys across 15 years to describe reductions in adolescent (aged 14 to 17) reports of supply of current alcohol by parent and friends’ since 2007. There was also an approximate halving of the rates at which adolescents reported parental supply of their first alcoholic beverage (26.5 % for 1998 participants compared to 13.9 % for 2013 participants). There were reductions in the rates of friends’ supply of alcohol in the 2013 surveys (both first ever alcoholic beverage and currently consumed alcohol), and there were reductions in the self-reported rates of purchasing alcohol. There were downward trends in most measures of alcohol use, with reductions falling to below 1998 rates in 2010. The rates of heavy alcohol use also fell to below 1998 rates from 2004 onwards. These results are good news for stakeholders in adolescent health.

The causal role of legislative and other interventions is impossible to determine from survey data. Results do point to several feasible links between alcohol supply sources and adolescent alcohol use. From 2007, reductions in parental supply of alcohol coincided with reductions in two of the three indices of adolescent alcohol use (weekly alcohol use and lifetime abstinence). This was also the case for heavy alcohol use but it was clear that heavy alcohol use was on a strong downward trend prior for the surveys prior to 2007. These findings are consistent with the possibility that parental supply rates may have a stronger impact on the initiation of alcohol use than on heavy alcohol use, and that other factors are likely to have contributed to reductions in heavy alcohol use. The findings in relation to changes in alcohol use are consistent with other Australian research that parental supply increases rather than decreases alcohol consumption [14]. It is also in line with Australian and American longitudinal research showing that adult-supervision of adolescent alcohol use is associated with increased alcohol use and alcohol-related harms [18].

Our findings suggested that the decrease in parental supply was not compensated for by other sources of alcohol. Over 2010 and 2013, there was no corresponding increases in rates of obtaining alcohol from these other sources. Rather, the results indicated a general reduction in adolescent alcohol use, which is consistent with downward trends in their major potential sources of alcohol. Our findings are also consistent with the possibility that cultural changes in adolescent alcohol use in Australia have reduced pressure on parents to provide alcohol, at least in the early phases of alcohol use. Further research is needed on changing attitudes towards alcohol use, alcohol misuse and health enhancement and risk amongst young Australians.

There is a need for further research on the impact of legislative changes with respect to supply of alcohol to people under 18 years of age (minors). In Australia, there has been considerable variability in the timing and nature of legislative changes on parental supply across Australian States and Territories. In the Australian Capital Territory (ACT), Western Australia (WA) and South Australia (SA), secondary supply of alcohol consumed in private residences is unregulated [21]. It is an offence for an individual other than a parent or guardian or authorized person to supply alcohol to a minor on private premises in New South Wales (NSW, introduced 2007), Northern Territory (NT, introduced 2011), Queensland (QLD, introduced 2009), Tasmania (TAS, introduced 2009) and Victoria (VIC, introduced 2011) [21]. Of the five Australian jurisdictions that have secondary supply legislation, three (NT, QLD and TAS) have legislated that alcohol consumption by a minor needs to be supervised by the responsible parent or guardian who supplied the alcohol [21]. There are two basic challenges in exploring links between the passage of parental supply legislation and adolescent alcohol use. First, there is considerable variation in the timing, extent and content of parental supply legislation between different states and territories. Second, it is unclear to what extent governments have been effective in getting public health messages across to parents about supplying alcohol. A national prevention trial targeting parental supply is currently underway in Australia [24] and should provide clearer evidence on the impact of reduced availability of alcohol on adolescent alcohol use.

Despite its nationally representative nature and the coincidence of reduced parental supply of alcohol and some measures of adolescent alcohol use, the cross-sectional data preclude strong inferences about causal factors. Parental supply may have reduced adolescent alcohol use, or parents may have experienced less pressure to supply alcohol to their adolescent children, or the two factors may be epiphenomenal to other cultural changes in adolescent health behavior. The present study did not control for the density of liquor outlets, which is a known associate of adolescent alcohol use [23]. There were also a range of other potential parental influences on adolescent alcohol use which were not assessed, including parents’ own alcohol use, general discipline, and parent–child relationship quality [25]. We could not examine parental supply of alcohol in supervised versus unsupervised settings, which is associated with variation in risky adolescent alcohol use [9]. The surveys depend on the reliability of adolescent reports and while there were changes to data collection methods that coincide with reductions in parental supply, this downward trend was consistent with findings in representative school surveys of adolescent alcohol use [29]. The same conclusion was also reached from comparisons between the drop-and-collect subsamples in 2007, and 2010 and 2013 (which only used drop-and-collect surveys).

## Conclusion

In Australia there have been recent reductions in the rates of parental supply of alcohol to adolescents. It remains unclear to what extent legislative changes in parental supply and broader cultural changes were responsible for reductions in parental supply and adolescent alcohol use.

### Ethics approval and consent to participate

The surveys were approved by the Australian Institute of Health and Welfare Ethics Committee. Access to these survey data by the Centre for Youth Substance Abuse Research was approved by the Australian Social Science Data Archive and by The University of Queensland Human Research Ethics Committee. Signed parental consent was obtained for adolescents under 16 years of age. If participants were 16 years old or older, signed consent was not required, and participants were taken to have given informed consent via letter/brochure scaffolding and completion of the questionnaire.

### Consent for publication

Not applicable.

### Availability of data and materials

The datasets on which these findings are available through the Australian Institute of Health and Welfare.

## Abbreviations

ACT: Australian Capital Territory
NDSHS: national drug strategy household survey
NSW: New South Wales
NT: Northern Territory
QLD: Queensland
SA: South Australia
TAS: Tasmania
VIC: Victoria
WA: Western Australia
